# 

**Published:** 1827-06

**Authors:** 


### MONTHLY LIST OF MEDICAL BOOKS.

[*No books can be entered on this List except those sent to us for the purpose; as, in the list hitherto transmitted, the names of works have frequently been given as published, which have not appeared for weeks, or even months, after.*]

Pathological and Practical Observations on Spinal Diseases: illustrated with Cases and Engravings. Also, an Inquiry into the Origin and Cure of Distorted Limbs. By EDWARD HARRISON, M.D. F.R.A.S. ED.; formerly President of the Royal Medical and Royal Physical Societies of Edinburgh, &c. &c.—Royal 8vo. pp. 294; with Engravings. London, 1827.

An Essay on Gout: in which its actual Predisponent, Proximate, and Exciting Causes are clearly defined; and its Preventive and Curative Indications fully demonstrated, upon new Pathological Principles, which exhibit a more consistent, safe, and efficient Method of Treatment than any hitherto promulgated. To which are added, Observations on the Modus Operandi of Bath Waters in Gouty Habits. By P. P. P. MYDDELTON, M.D. &c. Author of a Treatise on the Diagnosis and Prognosis of Diseases; Clinical Reports of Select Medical Cases, with Practical Illustrations; and a new System of Pulmonary Pathology. Fourth Edition.—8vo. pp. 97. Bath, 1827.

A Treatise on the Nature and Cure of Rheumatism; with Observations on Rheumatic Neuralgia, and on Spasmodic Neuralgia, or Tic Douloureux. By CHARLES SCUDAMORE, M.D. F.R.S. Honorary Member of Trinity College, Dublin; Physician in Ordinary to his Royal Highness the Prince Leopold of Saxe Coburg, &c. &c.—8vo. pp. 589. London, 1827.

Morborum Definitiones Causæque Continentes, &c. &c. quibus accedit Toxicologia. Auctore RICARDO MADDOCK HAWLEY, M.D. Collegii Regii Medicorum Edinensis Socio, &c.—8vo. pp. 364. Edinburgh, 1827.

A Treatise on Clinical Medicine, being a Compendious and Systematic Introduction to Practice, as contained in the Memoranda of I. R. BISCHOFF, M.D. Imperial Professor of Clinical Medicine, Physician to the General Hospital, and also to the Lying-in Hospital in Prague. From the German, by JOSEPH COPE, M.D.—12mo. pp. 280. London, 1827.

Clinical Observations on the Efficacy of the Hydrochloruret of Lime, as a Remedy in certain Stages of Fever and Dysentery. By ROBERT REID, M.D. Author of a Treatise on Tetanus and Hydrophobia, &c. &c.—8vo. pp. 35. Dublin, 1827.

The Hunterian Oration, delivered before the Royal College of Surgeons in London, on Wednesday, February 14, 1827. By H. LEIGH THOMAS, F.R.S. Member of the Imperial Academy at St. Petersburg, &c.—4to. pp. 28. 1827.

An Oration delivered before the Medical Society of London, on Thursday, March 8, 1827, (being the Fifty-fourth Anniversary,) by WM. KINGDON, Vice-President, &c. &c.—8vo. pp. 16. London, 1827.

Observations on the Necessity of establishing a different System of affording Medical Relief to the Sick Poor, than by the Practice of Contracting with Medical Men, or the Farming of Parishes. By J. F. HULBERT, M.R.C.S. &c. Melksham, Wilts.—8vo. pp. 51. Shrewsbury, 1827.

An Account of the Apparatuses for the Treatment of Rheumatism and Diseases of the Skin, which have been constructed at the Dublin Skin Infirmary; illustrated by many Plates. By Wm. Wallace, m.r.i.a. Surgeon to the Charitable Infirmary of Dublin, and to the Infirmary for the Treatment of Rheumatism and Cutaneous Diseases in that City, &c. &c. Second Edition. —4to. pp. 44. Dublin, 1827.

Some Account of the Science of Botany; being the Substance of an Introductory Lecture to a Course on Botany, delivered in the Theatre of the Royal Institution of Great Britain. By John Frost, f.a.s. f.l.s. &c. &c. (Dedicated by permission to the King.)—4to. pp. 17. London, 1827.

Medical Botany, Nos. IV. and V.; containing Conium Maculatum, Citrus Aurantium, Olea Europœa, Anagallis Arvensis, and Solanum Dulcamara, Digitalis Purpurea, Paris Quadrifolia, Tussilaga.—We always have pleasure in looking over the Numbers of this work: the Plates are really fine specimens of the art; nothing can exceed the accuracy with which most of them are executed,—the Anagallis for example. We think the veins on the leaf of the Paris Quadrifolia too strongly marked. By the by, this rare plant, in addition to the places mentioned, grows also at a spot called the "Corby Pot," near Aberdeen, where we have ourselves gathered it.

### METEOROLOGICAL JOURNAL,

### NOTICES.

*Communications have been received from* Dr. Ridgway, Mr. D. Fox, Mr. Frost, Mr. P. Blackett, Mr. C. Williams, Mr. Walsh, Mr. G. Bennett, *and from a Correspondent in Alcester, whose name we cannot decipher.*

### INDEX

TO THE

FIRST VOLUME OF THE NEW SERIES

OF THE

### London Medical and Physical Journal.

PAGE

Acephalous Fœtus, case of . 135
Acupuncture in Neuralgia . 91
Amaurosis cured by Tartar Emetic . . . . 564
Amputating saw, improved . 379
Amputation of the Penis, mode of avoiding . . . 374
Anatomical preparations, directions for making, in hot climates 558
Anatomy and diseases of the Nail, observations on the . . 289
Aneurism from bleeding . 147
———, dissection of a case in which the carotid artery was supposed to be tied . . 376
———, diffused, observations on 498
——— of the Subclavian, successfully treated by ligature . 502
——— ditto, unsuccessful, with some unusual appearances . 504
———, Popliteal, case of, with the state of the vessel two years after . . . 506
———, observations on, particularly with regard to the method of applying a ligature beyond the tumor . . 509
Antimony, Tartarised, its use in Gastro-enteritis . . 466
Aorta, obliteration of, case of the 280
Apology from the Editor of the Edinburgh Journal of Medical Science . . . . 189
Arsenic, inefficiency of vomiting in removing it from the stomach 246
Arteries, cases of wounded and diseased, with practical remarks . . 233, 327
Artery, Brachial, wound of . 518
Artimesia, its use in Epilepsy . 465
Asthma, cured by blowing air into the lungs . . 564

Bark, use of, in Iritis . . 476

PAGE

Bell, Mr.—*review of* his Appendix to the Papers on the Nerves 248
Belladonna, two cases of, poisoning by . . . 376
———, Mr. Blackett on, *review of* . . . 73
———, case of constitutional affection from external use of 286
Bitter almonds, case of a man who died from eating . . 150
Bladder, cases of rupture of the 417
Bones, syphilitic pains and diseases of the . . . . 303
Books, lists of, 95, 191, 287, 383, 479, 575
Botany, Medical, review of . 274
Bronchitis, use of Copaiba in . 491

Carotid Aneurism, dissection of a supposed case of . . 376
Cantharides, Tincture of, for the bite of venomous serpents . 88
Castor-oil, acids discovered in . 472
Cellular membrane, induration of 137
——— inflammation, observations on . . . . 1
Cephalo-spinal fluid, account of 462
Children, peculiar sore-throat affecting . . . . 90
Chlorate of Soda, M. Labarraque on, *review of* . . . 75
Chorea, fatal case of . . 240
Clinical medicine, suggestions for the improvement of . . 357
Cold in nervous irritation, efficacy of . . . . 180
Communication between the Lymphatics and Veins . 79
Conglobate glands, structure and economy of . . . 97
Consumptive patients, inexpediency of sending them to Madeira . . . . . 564
Copaiba, use of, in Bronchitis . 491

PAGE
Cornea, ulceration of, from inanition . . . . 276

Dentition, case of triple . 372
Depilation, case of spontaneous 275
Derangement of the Mind, *review of* Dr. Knight on . 454
Dissection, case of wound received in . . . 142
——— of one of the cases in which the Carotid Artery was supposed to be tied beyond the tumor . . . 376
Distortion, on the different modes of treating . . . 211
Dropsy, case of, treated with Kino . . . . 133
Drowned, mode of restoring those apparently . . . 375

Emphysema, case of . . 26
Epilepsy, case of, cured by Galvanism . . . 563
———, use of Artemesia in . 465
Epistaxis, method of arresting . 470
Equisetrum, diuretic properties of . . . . 182
Erosion, gangrenous, in children 523
Eruptions, on the suppression of, in children . . . 527
Erysipelas, cases of, accompanied by affection of the throat . 193
Exhumation, case of, three years after the interment . . 467

Fœtus, extra-uterine, case of . 378
Forceps, improved, for the use of dentists, &c. . . . 555
Fractures, on the treatment of, with description of an apparatus for suspending the limb . 229
Fumigations, remarks on . 283

Galvanism, Mr. La Beaume on, *review of* . . . 69
Gangrenous erosion in children 523
Gonorrhœa, combination of Cubebs and Copaiba in . 470
Gravel, two new kinds of . 464

Head, injuries of, at the Middlesex Hospital, 15, 111, 117, 242, 335
Hemorrhage, observations on . 569
Hernia, Inguinal, case of, with remarks . . . 52
———, singular variety of . 529
Hydatids in the Tibia, case of . 530
Hypertrophy of the Heart, case of . . . . 376

PAGE
Hypertrophy of the Heart, case of . . . . 489

Ice, impropriety of applying it to the head in cases of cerebral inflammation . . 373
Inflammation, observations on . 281
Indigestion, *review of* Dr. W. Philip on the more protracted cases of . . . 435
Injuries of the Head, series of cases of . 15, 111, 117, 242, 335

Lancets, sharp or blunt, in vaccination . . . 475
Laryngotomy, observations on . 566
Laurel-water in Epilepsy . 89
Leeches, application of . 573
Lexicon Pharmacopœium . 79
Lightning, peculiar effects of . 474
Luminousness of the eyes of animals . . . . 175
Lunar Caustic, directions for its application . . . 322

Materia Indica, Dr. Ainslie on, *review of* . . . 77
Measles, successful inoculation of 179
Mercurial frictions in Puerperal Peritonitis . . . 372
Meteorological Journals, 96, 192, 288, 384, 479, 576
Milk, metastasis of . .
Monthly Reports of prevalent Diseases, 92, 183, 279, 375, 475, 573
——— List of Medical Books, 95, 191, 287, 383, 479, 575

Nails, *review of* M. Collard on Alterations of the . . 267
———, anatomy and diseases of . 289
Necrology . . . 94
Nerves, Mr. Bell's Appendix to Papers on the, *review of* . 218
———, cases illustrating pathologically the functions of the 413
Neuralgia, cases of, cured by Carbonate of Iron . . 477
Notices to Correspondents, 96, 480, 576

Obesity, case of, cured by Iodine 465
Obituary . . . 479
Offal, peculiar local affection produced by . . . 342
Ophthalmia, Rheumatic . . 37
———, Catarrho-Rheumatic 292
Opium and its constituents, action of . . . . 561

PAGE
Paraplegia, inquiry into the cerebral origin of . . 385
————, facts relative to . 392
Parietal bones divided by an additional suture . . 175
Pericardium, case where it was tapped . . . 470
Pharmacopœia, Mr. Rennie's Supplement to the, *review of* . 78
Phenomena of Inflammation, remarks on the . . . 281
Physiology, *review of* Mr. Mayo's, 345
Penis, mode of avoiding the amputation of . . . 374
Pleximetre, account of the . 473
Poisoned wounds, M. Bouillaud on . . . . . 84
———— sugar-plums . 473
Pregnancy, case of tapping in the sixth month of . . 430
Prize questions . . . 475
Pulmonary complaints, remarks on . . . . 465
Pyloris, case of bony tumor obstructing the . . . 433

Quina, Sulphate of, in friction . 90

Reconciliation between Mr. J. H. Green and Mr. B. Cooper 376
Rectum, injury of the . . 90
Report of prevalent Diseases, 92, 183, 279, 375, 475, 573
Répertoire général d'Anatomie, &c. *review of* . . . 365
Rheumatism, acute, remarks on 123
———— and some Diseases of the Heart, *review of* Dr. Hawkins on . . . 260
Rhubarb, natural history of . 472

Salivation from mercurial vapour 282
Schirrus, cases of . . 129
Singultus, severe case of . 466
Small-pox, case of, twenty-three years after vaccination . 410
———— as it prevailed at Bury St. Edmund's . . . 406
———— after Vaccination, proportion of cases of . . 408
Stethoscope, cases illustrating the use of the . . . 481

PAGE
Stomach, ulcer of . . 24
————, Morbid Sensibility of, *review of* Dr. Johnson on . 58
Stomach-pump, application of, in intoxication . . . 574
————, improved form of . . . . 379
Stramonium, cases of poisoning by . . . . 563
Strictures of the Urethra, *review of* Mr. Andrews on . . 446
Sugar-plums, poisoned . 473
Syphilitic pains and diseases of the bones . . . 303

Tapping the Pericardium, case of 470
Tartar Emetic, its use in Gastroenteritis, &c. . . 466
Tea, green, effects of . . 570
Tetanus, acute Traumatic, case of 319
Throat, peculiar affection of, in children . . . 86
Transfusion, successful case of . 184
Tumor in the Spermatic Cord, resembling incarcerated Hernia 237
———— in the Abdomen . 464

Urethra, cases of ruptured 44, 46
Urinary gravel, two new kinds of 464
———— calculi, Mr. Wood on 29
———— organs, cases of diseases of the . . . 421
Urine, time required by various substances to manifest their presence in the . . 178

Vaccination, Mr. North on . 93
————, Dr. Gregory on 187, 400
————, Mr. Greenhow on . 188
Vaccine Board, their Annual Report . . . 383
———— vesicles, the effect of applying cupping glasses over . 462
Variola, M. Ribes on . . 85
Varioloid and vaccine virus, identity of the . . . 463
Vertebral column, two new articulations in the . . 175
Vomiting, inefficiency of, in removing arsenic from the stomach 246

### ORIGINAL PAPERS AND CASES.

PAGE

### INJURIES OF THE HEAD.

Cases of Injuries of the Head, treated at the *Middlesex Hospital*, by Mr. C. Bell, Mr. SHAW, and Mr. JOBERNS . 15, 111, 242, 335
——— Injury of the Head. By Mr. BOYLE, Surgeon to the *Middlesex Infirmary* . . . . . . . . . 117

### DISEASES OF THE CHEST.

Cases and Observations illustrating the Use of the Stethoscope. By Dr. M'ANDREW, (*South London Dispensary*) . . . . . 481
Case of Hypertrophy of the Heart, with Remarks. By Dr. MILLIGAN, (*Middlesex Infirmary*) . . . . . . . . 489
Cases and Remarks illustrating the Use of the Balsam of Copaiba in Bronchitis. By Dr. LA ROCHE . . . . . . 491

### ANEURISM.

Cases of Aneurism from Bleeding, which were treated at *St. George's Hospital*, under the care of B. C. BRODIE, Esq. . . . 147
Dissection of one of the Cases of Aneurism in which the Carotid Artery was supposed to be tied beyond the Tumor . . . . . 376
Observations on Diffused Aneurism; by Mr. DICKINSON, (*Macclesfield Dispensary*) . . . . . . . . . 498
Successful Case of Ligature of the Subclavian Artery; by Dr. ARENDT, (*Artillery Hospital, St. Petersburgh*) . . . . . . 502
Case of ditto, with some unusual Post-mortem Appearances; by Mr. BRODIE, (*St. George's Hospital*) . . . . . . 504
——— Popliteal Aneurism, with the State of the Vessels two years after the Operation; by Mr. SIMPSON, (*Regimental Hospital, Coldstream Guards*) . . . . . . . . . 506
Observations on Aneurism, particularly with regard to the Method of applying a Ligature beyond the Tumor; by Mr. SHAW, (*Middlesex Hospital*) . . . . . . . . . 509

### WOUNDED AND DISEASED ARTERIES.

Cases of Wounded and Diseased Arteries, treated principally at *St. Thomas's Hospital*, by B. TRAVERS, Esq. F.R.S. . . 233, 327
Case of Obliteration of the Aorta . . . . . . . 280
——— Wound of the Brachial Artery, with Observations. By Mr. WHITE, (*Westminster Hospital*) . . . . . . 518

### URINARY ORGANS.

Observations on the Analysis of Urinary Calculi, particularly those of a mixed nature. By JOHN F. WOOD, Lecturer on Chemistry . . 29
Cases of Rupture of the Bladder. By Messrs. BELL and SHAW, (*Middlesex Hospital*) . . . . . . . . . 417
——— Disease in the Urinary Organs. By Mr. JEFFREYS, (*St. George's Hospital*) . . . . . . . . . . 421

### RUPTURE OF THE URETHRA.

Case of Rupture of the Urethra, without external Wound, occasioned by falling across a wooden Railing. Treated by Mr. TRAVERS, at *St. Thomas's Hospital* . . . . . . . . . 44
——— Laceration of the Urethra, without external Wound, occasioned by falling across the edge of a Boat. Treated by Mr. GREEN, at *ditto* 46

PAGE

### HERNIA.

Case of Strangulated Oblique Inguinal Hernia, in which several of the Diagnostic Signs were wanting; with Clinical Remarks. Treated by Mr. Tyrrell, at *St. Thomas's Hospital* . . . . . 52
——— a singular Variety of Hernia, treated by Mr. Brodie . . 529

### OPHTHALMIA.

Practical Observations on Rheumatic Ophthalmia; with Cases. By Wm. Mackenzie, Andersonian Professor of Anatomy and Surgery, and one of the Surgeons to the *Glasgow Eye Infirmary* . . . . . 37
——————— Catarrho-Rheumatic Ophthalmia; with Cases. By Wm. Mackenzie, &c. . . . . . . 292

### DROPSY.

Case of Dropsy. By Dr. Paul, of Elgin . . . . . 133
——— Tapping in the sixth Month of Pregnancy. By Mr. Russell 430

### PARAPLEGIA.

An Inquiry into the alleged Cerebral Origin of certain Cases of Paraplegia. By Thomas Harrison Burder, m.d. . . . 385
Facts relative to Paraplegia. By the late Dr. Baillie . . 392

### ERYSIPELAS.

Cases of Erysipelas accompanied by Affection of the Throat: with Remarks on the Propriety of limiting the Application of the Term. By James M. Arnott, Esq. Surgeon . . . . . . 193

### RHEUMATISM.

Remarks on Acute Rheumatism; with Cases. By Anthony Todd Thomson, m.d. . . . . . . . . 123

### DISTORTION OF THE SPINE.

On the different Modes of treating Distortion of the Spine. By John Shaw, Esq. . . . . . . . . . 211

### FRACTURES.

On the Treatment of Fractures of the Bones of the Lower Extremity; with a Description of an improved Apparatus for suspending the Limb. By William Chandler, Esq. . . . . . . 229

### SMALL-POX AND VACCINATION.

Note on Vaccination, by Dr. Gregory . . . . . . 187
——————, by Mr. Greenhow . . . . . 188
On the Permanent Evidences of successful Vaccination. By Dr. Gregory 400
On Small-Pox as it prevailed epidemically at Bury St. Edmund's. By Mr. Dalton . . . . . . . . 406
Proportion of Cases of Small-Pox after Vaccination. By E. Morton, m.b. l.m. . . . . . . . . . 408
Case of Small-Pox twenty-three years after Vaccination. By Dr. Heineken . . . . . . . . . 410

### DISEASES OF CHILDREN.

Case of Induration of the Cellular Membrane in a Child. Treated by Dr. M'Andrew, at the *South London Dispensary* . . . . . 137
Observations on Gangrenous Erosion in Children, by E. Thompson, Esq. 523
On the Suppression of Cutaneous Eruptions in Children, by E. Morton, m.d. *(Metropolitan Infirmary for Children)* . . . . 527

PAGE

### MISCELLANEOUS.

Observations on diffused Cellular Inflammation; with Cases. By HENRY EARLE, Esq. F.R.S. &c. *(St. Bartholomew's Hospital)* . . 1
Case of Ulcer of the Stomach. By Mr. HUNTER . . . 24
——— Emphysema. By Mr. SYM . . . . . 26
Observations on the Interior Structure and Economy of the Conglobate Glands. By JOHN CHARLES OGILVIE, M.D. . . . 97
Case of Congenital Fissure of the Soft Palate. By HERBERT MAYO, Esq. 119
Cases showing the Constitutional Predisposition to the Formation of Schirrus in different parts of the Body at the same time. By Mr. JEFFREYS, Surgeon to *St. George's Hospital* . . . . 129
Description of an Acephalous Fœtus. By ROBERT ABRAHAM, Esq. . 135
Case of Injury received in Dissection. By JOHN SHAW, Esq. . 142
——— a Man who died from eating to excess of the Bitter Almond. By Mr. KENNEDY. With some Remarks, by Dr. PARIS . . 150
——— Uterine Hemorrhage, successfully treated by Transfusion. By Mr. BROWN . . . . . . . . . 184
Account of a Case, in which a Tumor in the Spermatic Chord was complicated with Symptoms so strongly resembling those of Incarcerated Bubonocele, as to lead to an Operation, by which the true Nature of the Disease was ascertained. Treated by HENRY JEFFREYS, Esq. . 237
Fatal Case of Chorea, treated at the *Middlesex Hospital*, by Dr. HAWKINS 240
On the Inefficiency of the Act of Vomiting in removing Arsenic from the Stomach. By JAMES SCOTT, Surgeon . . . . 246
Remarks on some of the Phenomena of Inflammation. By Mr. WISE 281
Salivation speedily produced by inhaling Mercurial Vapour. By Mr. SOMERVILLE . . . . . . . . . 282
Practical Remarks on the Utility of Fumigations. By Mr. GREEN . 283
Case in which Constitutional Effects arose from the external Application of Belladonna. By Mr. WADE . . . . . . 286
Observations on the Anatomy and Diseases of the Nails. By Sir ASTLEY COOPER, Bart, &c. &c. . . . . . . . 289
On Syphilitic Pains and Diseases of the Bones. By C. HAWKINS, Esq. 303
Case of Acute Traumatic Tetanus; with some Observations, as given in a Clinical Lecture by H. EARLE, Esq. *(St. Bartholomew's Hospital.)* . 319
Directions for using the Lunar Caustic. By J. HIGGINBOTTOM, Esq. 322
Cases illustrating the History of a peculiar Local Disease, apparently produced by the Application of a Poisonous Matter contained in Offal. By B. C. BRODIE, Esq. F.R.S. &c. . . . . . . 342
Two Cases of Poisoning by Belladonna. By Mr. SMITH, Surgeon . 376
Extra-Uterine Fœtus . . . . . . . . 378
Improved Stomach-Pump, and Amputating Saw. By Dr. FOX . 379
Cases pathologically illustrative of the distinct and separate Nervous Functions subservient to Voluntary Motion and Feeling. By Mr. BROUGHTON . . . . . . . . . 413
Case of a Bony Tumor obstructing the Pylorus. By Dr. WEBSTER . 433
Use of Bark in Iritis. By Mr. WALLACE . . . . 476
Cases of Neuralgia, by Dr. DARWALL and Mr. WICKENDEN . . 477
Case of Hydatids in the Tibia, by Mr. WICKHAM, *(Winchester Hospital)* 530
On the Application of Leeches. By S. G. LAWRANCE, Esq. . . 573
Application of the Stomach-Pump in a Case of Intoxication. By J. HARRISON, Esq. . . . . . . . . 574

### CRITICAL ANALYSES.

PAGE

An Essay on the Morbid Sensibility of the Stomach and Bowels, as the Proximate Cause, or Characteristic Condition, of Indigestion, Nervous Irritability, &c. &c. By JAMES JOHNSON, M.D. . . . . 58
On Galvanism. By Mr. LA BEAUME . . . . . . 69
An Essay on the Use of the Atropa Belladonna. By Mr. BLACKETT . 73
The Use of the Chlorate of Soda, and the Chlorate of Lime. By M. A. G. LABARRAQUE . . . . . . . . 75
Materia Indica. By WHITELAW AINSLIE, M.D. M.R.A.S. &c. . . 77
A New Supplement to the Pharmacopœias, &c. By J. RENNIE, A.M. 78
Lexicon Pharmacopœlium, or a Pharmacopœial Dictionary. By Mr. CASTLE . . . . . . . . . 79
A Treatise on the Diseases of Children, &c. By the late MICHAEL UNDERWOOD, M.D. With Notes and Observations, by SAMUEL MERRIMAN, M.D. F.L.S. . . . . . . . 152
Nosological Practice of Physic, embracing Physiology. By Dr. DAWSON 159
An Oration, delivered before the Hunterian Society. By Sir WILLIAM BLIZARD, Knt. . . . . . . . . 168
Observations on the Artificial Mineral Waters of Dr. STRUVE. By Dr. KING . . . . . . . . . . . 172
Appendix to the Papers on the Nerves, republished from the Royal Society's Transactions, by CHARLES BELL; containing Consultations and Cases illustrative of the Facts announced in these Papers . 248
Rheumatism, and some Diseases of the Heart and other internal Organs. By FRANCIS HAWKINS, M.D. . . . . . . 260
On the Alteration of the Nails, and Disease of the surrounding Skin. By M. HIPP. ROYER COLLARD . . . . . . . 267
Medical Botany; or, Illustrations and Descriptions of the Medicinal Plants of the London, Edinburgh, and Dublin Pharmacopœias. Nos. I. and III. . . . . . . . . . 274
Outlines of Human Physiology. By HERBERT MAYO, Esq. &c. . 345
Observations on the System of teaching Clinical Medicine in the University of Edinburgh, with Suggestions for its Improvement. By JAMES CLARK, M.D. . . . . . . . . . 357
Répertoire général d'Anatomie et de Physiologie, &c.
M. BRESCHET on Ectropium of the Organs of the Circulation, &c. 365
M. DUPUYTREN on the Displacement of the Head of the Femur . ib.
On the Treatment of the more protracted Cases of Indigestion. By Dr. WILSON PHILIP . . . . . . . . . 435
Practical Observations on the Application of Lunar Caustic to Strictures in the Urethra, &c. &c. By Mr. ANDREWS . . . . . 446
Observations on the Causes, &c. of Derangement of the Mind. By Dr. KNIGHT . . . . . . . . . . . 454
The Life of EDWARD JENNER, M.D. LL.D. F.R.S. &c. By Dr. BARON 532
A Critical Analysis of Dr. BARRY'S Memoir on Atmospheric Pressure being the principal Cause of the Progression of the Blood in the Veins. By Mr. SEARLE . . . . . . . . . 545
Observations on the Circulation of the Blood. By Dr. ARNOTT . 548
———————— Treatment of Gonorrhœa; with Cases. By Mr. THORN 552
Mr. FAY on an improved Forceps for the Use of Dentists and others . 555
Dr. ROGET on Human and Comparative Physiology . . . . 557

---

COLLECTANEA . . *Pages* 79, 175, 275, 372, 462, 558
INTELLIGENCE . . . 92, 183, 279, 375, 475, 573
METEOROLOGICAL REGISTER 96, 192, 288, 384, 480, 576

### NAMES OF AUTHORS,

*Of whose Works, Observations, &c. either a detailed Account, or more or less general View, is given in the present Volume.*

PAGE

ABRAHAM, Mr. his case of Acephalous Fœtus . . . . . 135
ANDREWS, Mr. *review of*, on Strictures of the Urethra . . 446
AINSLIE, Dr. his Materia Indica . . . . . . . 77
ARNOTT, Mr. on Erysipelas . . . . . . . 193
——, Dr. his successful case of Ligature of the Subclavian . 502
—— his observations on the Circulation, as connected with Dr. BARRY's theory . . . . . . . . 548
BALLINGALL, Dr. his observations on Laryngotomy . . . 566
—— Hemorrhage . . . 569
BAILLIE, Dr. Facts relative to Paraplegia, from a posthumous MS. . 392
BARON, Dr. *review of* his Life of JENNER . . . . 532
BELL, Mr. C. his cases of Injury of the Head 15, 111, 117, 242, 335
—— Ruptured Bladder . . . . 417
BLACKETT, Mr. on Belladonna, *review of* . . . . 73
BLIZARD, Sir WM. his Oration before the Hunterian Society, *review of* 168
BOYLE, Mr. his case of Injury of the Head . . . . 117
BRODIE, Mr. his cases of Aneurism . . . . . 147, 504
—— his account of a peculiar Local Affection, produced by handling Offal . . . . . . . . . 342
—— his singular case of Hernia . . . . 529
BROUGHTON, Mr. his cases illustrating the Functions of the Nerves . 413
BROWN, Mr. his case of successful Transfusion . . . 184
BURDER, Dr. on Paraplegia . . . . . . . 392
CHANDLER, Mr. on the Treatment of Fractures of the Lower Extremities . . . . . . . . . . 229
CLARK, Dr. on Clinical Medicine, *review of* . . . . 357
COOPER, Sir ASTLEY, on the Nails . . . . . 289
—— Mr. B. his reconciliation with Mr. J. H. GREEN . . 376
COLLARD, M. *review of*, on the Nails . . . . . 267
DALTON, Mr. on Small-Pox . . . . . . . 406
DAWSON, Dr. his Nosological Practice of Physic, *review of* . . 159
DAVY, Dr. J. his directions for making Anatomical Preparations in hot Climates . . . . . . . . . . 558
EARLE, Mr. on Cellular Inflammation . . . . . 1
—— his case of Traumatic Tetanus . . . . 319
FAY, Mr. account of his improved Forceps . . . . 555
GREEN, Mr. his case of Ruptured Urethra . . . . 46
—— on Fumigations . . . . . . . 283
—— his reconciliation with Mr. B. COOPER . . . 376
GREGORY, Dr. on Vaccination . . . . . . 400
HAWKINS, Dr. his case of Fatal Chorea . . . . 240
——, *review of*, on Rheumatism, &c. . . . . 260
——, Mr. CÆSAR, on Syphilitic Pains and Diseases of the Bones . 303
HEINEKEN, Dr. his case of Small-Pox twenty-three years after Vaccination . . . . . . . . . . 410
HIGGINBOTTOM, Mr. on Lunar Caustic . . . . . 322
HUNTER, Mr. his case of Ulcer of the Stomach . . . . 24
JEFFREYS, Mr. his case of Schirrus . . . . . 129
—— Tumor resembling incarcerated Bubonocele 237
—— cases of Disease of the Urinary Organs . . . 421
JENNER, Dr. *review of* his Life . . . . . . 532
JOBERNS, Mr. his cases of Injury of the Head . . . 111, 335

PAGE
JOHNSON, Dr. on Morbid Sensibility of the Stomach, *review of* . 58
KENNEDY, Mr. his case of a Man who died from eating Bitter Almonds 150
KING, Dr. his observations on the Artificial Mineral Waters of Dr. Struve, *review of* . . . . . . . . . 172
KNIGHT, Dr. on Mental Derangement, *review of* . . . 454
LABARRAQUE, Mr. on Chlorate of Soda, *review of* . . . 75
LA BEAUME, Mr. on Galvanism, *review of* . . . . 69
M'ANDREW, Dr. his case of Induration of the Cellular Membrane . 137
————, cases illustrating the use of the Stethoscope . . 481
MACKENZIE, Mr. Professor, on Ophthalmia . . . 37, 292
MAYO, Mr. HERBERT, case of Congenital Fissure of the Soft Palate . 119
————, *review of* his Physiology . . . 345
MERRIMAN, Dr. his edition of Dr. UNDERWOOD on the Diseases of Children, *review of* . . . . . . 152
MILLIGAN, Dr. his case of Hypertrophy . . . . 489
MORTON, Dr. on Small-Pox after Vaccination . . . 408
———— on the Suppression of Eruptions in Children . . 527
OGILVIE, Dr. on the Conglobate Glands . . . . 97
PAUL, Dr. his case of Dropsy treated with Kino . . . 133
PHILIP, Dr. W. on the more protracted Cases of Indigestion . . 435
RENNIE, Mr. *review of* his Supplement to the Pharmacopœia . . 78
RENTON, Dr. on the Inexpediency of sending Consumptive Patients to Madeira . . . . . . . . 564
ROGET, Dr. notice of his Lecture on Human and Comparative Physiology 557
RUSSELL, Mr. his successful case of Tapping during Pregnancy . 430
SEARLE, Mr. *review of* his Critical Analysis of Dr. BARRY's Memoir . 545
SHAW, Mr. his case of Injury from Dissection . . . . 142
———— on the different Modes of treating Distortion of the Spine 211
———— observations on Aneurism . . . . . 509
SCOTT, Mr. on the Inefficacy of Vomiting in removing Arsenic from the Stomach . . . . . . . . . 246
SOMERVILLE, Dr. his case of Salivation following the Inhalation of Mercurial Vapour . . . . . . 282
SIMPSON, Mr. his case of Popliteal Aneurism . . . 506
SYM, Mr. his case of Emphysema . . . . . . 26
THOMSON, Dr. A. TODD, on Acute Rheumatism . . . 123
————, Mr. on the Gangrenous Erosion of young Children . 523
THORN, Mr. *review of* his Observations on Gonorrhœa . . 552
TRAVERS, Mr. his case of Ruptured Urethra . . . . 44
———— cases, &c. of Wounded and Diseased Arteries 233, 327
UNDERWOOD, Dr. on the Diseases of Children, by Dr. MERRIMAN, *review of* . . . . . . . . 152
WADE, Mr. his case of Constitutional Effects from the external Use of Belladonna . . . . . . . . 286
WALLACE, Mr. on the Use of Bark in Iritis . . . . 476
WEBSTER, Dr. his case of Bony Tumor in the Stomach . . 433
WHITE, Mr. his case of Wound of the Brachial Artery . . 518
WICKHAM, Mr. his case of Hydatids in the Tibia . . . 530
WISE, Mr. on Inflammation . . . . . . 281
WOOD, Mr. on Urinary Calculi . . . . . 29

### PLATES.

Mr. Mayo's Case of Congenital Fissure of the Soft Palate, *to face page* 119
Mr. Shaw on Distortion of the Spine . . . . . 211
Sir Astley Cooper on the Anatomy and Diseases of the Nails . . 289
Mr. Mackenzie on Catarrho-Rheumatic Ophthalmia . . . 292

J. and C. Adlard, Printers, Bartholomew Close.

